# Is the current UK guidance for treatment of co-occurring substance use and mental health problems being implemented in practice?

**DOI:** 10.1192/j.eurpsy.2025.666

**Published:** 2025-08-26

**Authors:** L. Goodwin, Z. Swithenbank, P. Irizar, K. Jackson, L. Halsall, J. Puddephatt, P. Parkes, C. Angus, A. Ushakova, J. Knight, F. Lobban, C. Drummond, A. O’Donnell

**Affiliations:** 1 Lancaster University, Lancaster; 2 University of Manchester, Manchester; 3 Newcastle University, Newcastle; 4 Edge Hill University, Ormskirk; 5 Expert Citizens, Stoke-on-Trent; 6 University of Sheffield, Sheffield; 7 King’s College London, London, United Kingdom

## Abstract

**Introduction:**

Substance use and mental health problems commonly co-occur. Yet, people experiencing both problems commonly face barriers to getting the support they need and experience increased physical morbidity and premature mortality, compared to those with only one problem. In the UK, current guidance on treatment of co-occurring alcohol and mental health problems exist which set out standards for ways of working with this population, and how and where treatments should be offered.

**Objectives:**

Through a secondary qualitative analysis and a systematic review, we aimed to determine the extent to which the UK guidance for care for people with co-occurring problems is being implemented.

**Methods:**

A secondary qualitative analysis was conducted of interview transcripts from people with co-occurring depression and hazardous/harmful alcohol (N = 39). In addition, a systematic review was conducted to identify studies published in the UK since 2017 which focueds on treatment for adults with co-occurring substance use and mental health problems. For both data sources, a deductive coding framework was developed based upon the UK guidance and a thematic analysis was applied.

**Results:**

The qualitative analysis identified four key themes from the service user perspective: Wrong doors and stigma; Responsibility and coordination of care; ‘Don’t discharge me’ and Impacts of gaps between services. The review identified three main themes: Challenges to care for co-occurring conditions; Integration of care; and System and structural level barriers. Both studies showed that making initial contact with services was challenging, as was continued engagement with treatment. Staff attitudes and knowledge were important, because the therapeutic alliance had a significant impact on treatment outcomes and recovery. Stigma, both intrinsic and extrinsic, was identified as a barrier to accessing, engaging with, and delivering support. Both studies found evidence of inconsistent treatment offerings and conflicting advice or support, especially around the most appropriate way to treat co-occurring conditions. Barriers and facilitators were evident across individual, organisational and systems levels. Findings indicate that people are often not receiving the care they need in the ways in which guidance recommends.

**Conclusions:**

The results suggest that despite existence of current guidance in the UK, implementation is inconsistent. This work has identified specific areas for improvement around access to treatment and a need for better coordination and integration of care. It highlights the need to explore how guidance can be better embedded in current practice to improve experiences and outcomes for this population.

**Disclosure of Interest:**

None Declared

